# Association of pre-pregnancy body mass index and first-trimester remnant cholesterol level with risk of large for gestational age

**DOI:** 10.7189/jogh.16.04315

**Published:** 2026-09-04

**Authors:** Lihua Lin, Sisi Zhang, Juan Lin, Xiaomei Wang

**Affiliations:** 1 Department of Healthcare, Fujian Maternity and Child Health Hospital, College of Clinical Medicine for Obstetrics & Gynecology and Pediatrics, Fujian Medical University, Fuzhou, PR China; 2 Department of Healthcare, Fujian Obstetrics and Gynecology Hospital, College of Clinical Medicine for Obstetrics & Gynecology and Pediatrics, Fujian Medical University, Fuzhou, PR China; 3 Department of Obstetrics, Fujian Maternity and Child Health Hospital, College of Clinical Medicine for Obstetrics & Gynecology and Pediatrics, Fujian Medical University, Fuzhou, PR China

**Keywords:** large for gestational age, body mass index, remnant cholesterol level, gestational diabetes mellitus

## Abstract

**Background:**

To evaluate the associations of pre-pregnancy body mass index (BMI) and first-trimester remnant cholesterol (RC) level with the risk of large for gestational age (LGA) infants.

**Methods:**

A prospective cohort study was conducted in Southeastern China, including 11,319 pregnant women. Logistic regression was used to examine the associations of pre-pregnancy BMI and RC level with LGA. Restricted cubic spline regression was applied to assess the dose-response relationship between RC level and LGA risk. Mediation analysis was performed to evaluate the potential mediation role of gestational diabetes mellitus (GDM) in the associations of pre-pregnancy BMI and RC level with LGA.

**Results:**

Compared with the reference groups, LGA risk was significantly higher in women with a pre-pregnancy BMI≥24kg/m^2^ (odds ratio (OR) = 1.60; 95% CI = 1.31 ~ 1.95) and those in the highest tertile of RC (OR = 2.09; 95% CI = 1.67 ~ 2.61). No significant multiplicative or additive interactions were observed between BMI and RC level on LGA risk. Gestational diabetes mellitus mediated 0.91% (RC) and 1.15% (BMI) of effects. Population-attributable risk estimates indicated that 6.69 and 19.68% of LGA cases could be attributed to high pre-pregnancy BMI and elevated RC levels, respectively.

**Conclusions:**

Elevated RC and pre-pregnancy BMI are independent LGA risk factors, with minimal GDM mediation. Managing lipid profiles, particularly RC, is important even in high-BMI women.

Large for gestational age (LGA) is defined as a neonatal birth weight equal to or greater than the 90th percentile for sex and gestational age based on standardised birth weight curve [1]. Excessive birth weight poses significant short- and long-term risks for both maternal and infant health. Mothers carrying LGA foetuses are at increased risk of caesarean section, prolonged labour, the birth canal trauma, and postpartum haemorrhage [2,3]. Meanwhile, LGA infants face higher rates of shoulder dystocia, neonatal hypoglycaemia, brachial plexus injury, skeletal injury, clavicular fracture, perinatal asphyxia, and mortality [4–7]. Furthermore, LGA is associated with long-term metabolic sequelae, including cardiovascular disease, type 2 diabetes mellitus, and obesity in adulthood.

Pregnancy represents a complex and highly coordinated physiological process influenced by numerous internal and external factors. A favourable intrauterine environment is essential for normal foetal growth, whereas suboptimal conditions can lead to aberrant foetal development. Maternal lipids play a crucial role in foetal growth [8], and elevated lipid levels during pregnancy may contribute to foetal overgrowth. Studies have shown that maternal low-density lipoprotein cholesterol (LDL-C), high-density lipoprotein cholesterol (HDL-C), and apolipoprotein A1 (ApoA1) are lower in pregnant women with LGA foetuses compared to those with appropriate grown foetuses [9]. Additionally, the risk of macrosomia or LGA is positively correlated with maternal triglycerides (TG) and total cholesterol (TC), and inversely associated with HDL-C concentrations[10–14]. While these traditional lipid parameters have been consistently linked to foetal growth, recent attention has shifted toward non-traditional markers such as remnant cholesterol (RC), which may offer more nuanced insights into lipid-related risk [15].

RC represents the cholesterol content within triglyceride-rich lipoproteins, including very low-density lipoprotein (VLDL), intermediate-density lipoprotein (IDL), and chylomicron remnants [16]. In recent years, RC level has emerged as an important marker in lipid management [17]. Epidemiological evidence indicates that elevated RC levels are significantly associated not only with diabetes mellitus but also with related comorbidities such as hypertension [18,19]. However, the role of RC in pregnancy and its relationship with foetal growth remains poorly understood. It is hypothesised that RC may serve as a valuable predictor of foetal overgrowth. Furthermore, although pre-pregnancy BMI is a well-established risk factor for LGA [20], its potential interaction with RC levels has not been thoroughly examined. Therefore, this hospital-based prospective cohort study aims to evaluate the individual and joint associations of first-trimester RC levels and pre-pregnancy BMI with LGA risk in a Southeast Chinese population. By elucidating the interplay between these factors, this research aims to inform more effective preventive strategies to improve pregnancy outcomes for mothers and infants.

## METHODS

### Study population

This analysis utilised data from a hospital-based prospective birth cohort study conducted at a maternity and child health hospital. Pregnant women attending their first antenatal care visit before 13 weeks of gestation and planning to delivery at the hospital were invited to participate. Participants were followed *via* structured questionnaires administered during each trimester and through their routine antenatal care visits. The cohort study was approved by the Ethics Committee of hospital (approve number:2019-200), and written informed consent was obtained from all participants upon enrolment.

Eligibility criteria for the current analysis included:

• singleton pregnancy;

• maternal age between 15 and 45 years;

• availability of complete baseline data, lipid profiles, and delivery outcomes;

• gestational age at delivery of 28 weeks or more.

Women with pre-existing severe cardiovascular, hepatic, renal, or autoimmune diseases were excluded, as were those with missing essential data.

Between 2020 and 2022, a total of 12,567 pregnant women who had their first prenatal visit before 13 weeks completed all prenatal visits and delivered at Fujian Maternity and Child Health Hospital. After applying the inclusion and exclusion criteria, 11,319 women were included in the final analysis. A flowchart detailing participant selection is presented in Figure S1 in the **Online Supplementary Document**.

### Blood sampling

Fasting venous blood samples were collected after an overnight fast of at least eight hours. Serum level of TC, TG, HDL-C, LDL-C were measured at the clinical biochemistry laboratory of Fujian Maternity and Child Health Hospital using standard enzymatic methods.

### Measurements and definitions

Baseline maternal characteristics, including maternal age, gravidity, parity, height, pre-pregnancy weight, gestational age, infant birth weight, delivery mode, and infant sex, were obtained from medical records. Pre-pregnancy BMI was calculated as self-reported pre-pregnancy weight (kg) divided by height squared (m^2^). According to the Chinese adult weight classification standards, participants were categorised as underweight (BMI<18.5 kg/m^2^), normal weight (18.5≤BMI<24 kg/m^2^), or overweight/obese (BMI≥24 kg/m^2^) [21]. The RC level (mmol/L) was calculated using the formula: RC (mmol/L) = TC (mmol/L)-HDL-C(mmol/L)-LDL-C(mmol/L) [22]. Gestational weight gain (GWG) was calculated as maternal weight at delivery(kg) and the pre-pregnancy weight (kg).

### Statistical analysis

Continuous variables are presented as mean ± standard deviation for normally distributed data or as medians (interquartile range) for skewed data. Categorical variables are expressed as numbers (percentages). Baseline characteristics were summarised according to tertiles of RC level (low, middle, high). Group differences were assessed using *t* tests or Mann-Whitney U tests for continuous variables and χ^2^ or Fisher exact tests for categorical variables. Logistic regression models were used to estimate odds ratios (ORs) and 95% confidence intervals (CIs) for LGA across RC tertiles and pre-pregnancy BMI categories, with the lowest RC tertile and normal-weight women (BMI = 18.5–23.9) serving as references. Three sequential models were constructed: Model 1 was the crude model; Model 2 adjusted for maternal age (continuous variable), ethnicity (Han or other), occupation (Yes/not), gravidity (1,2 or ≥3), parity (primiparous or multiparous), gestational age at delivery (continuous), infant sex (boy or girl), and gestational weight gain (continuous). Model 3 was further adjusted for baseline TG (continuous), HDL-C (continuous), and GDM (yes/no). The dose-response relationship between continuous RC levels and LGA risk was evaluated using restricted cubic splines with three knots in *R*, version 4.3.0 (Foundation for Statistical Computing, Vienna, Austria).

Stratified analyses were performed by pre-pregnancy BMI category to examine the association between RC level and LGA within each subgroup. Additive and multiplicative interactions between pre-pregnancy BMI (<18.5kg/m^2^, 18.5–23.9kg/m^2^, and ≥24.0 kg/m^2^) and RC tertiles were assessed. Multiplicative interaction was evaluated *via* the OR and 95% CI of the product term. Additive interaction was estimated using three metrics: the relative excess risk due to interaction (RERI), the attributable proportion due to interaction (AP), and the synergy index (SI). The absence of additive interaction is indicated by RERI = 0, AP = 0, or SI = 1. Conversely, RERI>0, AP>0, and SI>1 suggest that the combined effect of BMI and RC on LGA risk exceeds the sum of their individual effects.

Mediation analysis was conducted using the SAS macro (%mediation) by Valeri and VanderWeele to estimate the potential mediating roles of GDM [23]. Two separate mediation models were tested:

• with pre-pregnancy BMI as the exposure, GDM as the mediator, and LGA as the outcome; and

• with RC level as the exposure, GDM as the mediator, and LGA as the outcome (Figure S2 in the **Online Supplementary Document**).

The proportion mediated was calculated as the indirect effect divided by the total effect. Full mediation is suggested when the indirect effect is significant but the direct effect is not, whereas significant direct and indirect effects indicate partial mediation [23].

Population attributable fractions (PAFs) were calculated to estimate the proportion of LGA cases attributable to high pre-pregnancy BMI (≥24.0 kg/m^2^) and elevated RC levels (defined using the cutoff derived from the RC model: 0.98 mmol/L) within the study population. A two-tailed *P*-value <0.05 was considered statistically significant for all analyses. The analyses were conducted using SAS software, version 9.3 (SAS Institute, Cary, NC, US), *R* software, version 4.3.0 (R Foundation for Statistical Computing, Vienna, Austria), and SPSS software, version 21.0 (SPSS Inc., Chicago, IL, USA).

## RESULTS

### Descriptive characteristics of the study population

A total of 11,319 participants were included in the final analysis. Their baseline characteristics, stratified by RC tertiles, are summarised in **Table 1**. The overall mean maternal age was 30.2 years (standard deviation (SD) = 4.2), height was 159.9cm (SD = 5.7), pre-pregnancy weight was 53.3 kg (SD = 7.1), and pre-pregnancy BMI was 20.8 kg/m^2^ (SD = 2.6). Over 98% of participants were of Han ethnicity, and more than 50% were multiparous. Participants in the highest RC tertile were more likely to be of Han ethnicity, employed, have lower gravidity, deliver at a later gestational age, be diagnosed with GDM, and give birth to LGA infants (all *P* < 0.001).

**Table 1:** Baseline characteristics of participants stratified by remnant cholesterol (RC) tertiles

Characteristic	Total	Tertile 1 (RC<0.8mmol/L)	Tertile 2 (0.8≥RC<1.2, mmol/L)	Tertile 3 (RC≥1.2mmol/L)	*P*-value
Number of patients	11,319	3,757	3,712	3,850	
Maternal age in years (mean ± SD)	30.2 ± 4.2	30.4 ± 4.3	30.1 ± 4.2	30.2 ± 4.1	0.038
Maternal height in cm (mean ± SD)	159.9 ± 5.7	159.8 ± 5.6	159.9 ± 5.6	160.0 ± 5.8	0.581
Pre-pregnancy weight in kg (mean ± SD)	53.3 ± 7.1	53.2 ± 7.2	53.2 ± 6.9	53.4 ± 7.2	0.333
Pre-pregnancy BMI, in kg/m^2^, (mean ± SD)	20.8 ± 2.6	20.8 ± 2.7	20.8 ± 2.5	20.9 ± 2.7	0.429
Pre-pregnancy BMI in kg/m^2^, n (%)					
*<18.5*	1,963 (17.3)	671 (17.9)	633 (17.1)	659 (17.1)	
*18.5–23.9*	8,092 (71.5)	2,663 (70.9)	2,685 (72.3)	2,744 (71.3)	
*≥24*	1,264 (11.2)	423 (11.3)	394 (10.6)	447 (11.6)	
Ethnicity, n (%)					<0.001
*Han*	11,117 (98.2)	3,667 (97.6)	3,648 (98.3)	3,802 (98.8)	
*Other*	202 (1.8)	90 (2.4)	64 (1.7)	48 (1.2)	
Occupation, n (%)					<0.001
*Yes*	7,945 (70.2)	2,549 (67.8)	2,626 (70.7)	2,770 (71.9)	
*No*	3,374 (29.8)	1,208 (32.2)	1,086 (29.3)	1,080 (28.1)	
Gravidity, n (%)					<0.001
*1*	6,906 (61.0)	2,179 (58.0)	2,281 (61.4)	2,446 (63.5)	
*2*	3,214 (28.4)	1,148 (30.6)	1,055 (28.4)	1,011 (26.3)	
*≥3*	1,199 (10.6)	430 (11.4)	376 (10.1)	393 (10.2)	
Parity, n (%)					0.249
*Primiparity*	5,170 (45.7)	1,735 (46.2)	1,654 (44.6)	1,781 (46.3)	
*Multiparity*	6,149 (54.3)	2,022 (53.8)	2,058 (55.4)	2,069 (53.7)	
Delivery					
*Maternal weight at delivery in kg (mean ± SD)*	68.1 ± 8.5	68.2 ± 8.8	68.0 ± 8.6	68.0 ± 8.2	0.388
*Total gestational weight gain in kg (mean ± SD)*	14.8 ± 9.5	15.0 ± 9.7	14.8 ± 9.6	14.6 ± 9.4	0.164
*Gestational age in weeks (mean ± SD)*	38.6 ± 1.9	38.3 ± 2.1	38.6 ± 1.8	38.7 ± 1.8	<0.001
Gestational weight gain category, n (%)					0.659
*As recommended*	2,806 (24.8)	905 (24.1)	917 (24.7)	984 (25.6)	
*Below recommended*	2,883 (25.5)	959 (25.5)	955 (25.7)	969 (25.2)	
*Above recommended*	5,630 (49.7)	1,893 (50.4)	1,840 (49.6)	1,897 (49.3)	
GDM, n (%)					<0.001
*Yes*	1,864 (16.5)	559 (14.9)	609 (16.4)	696 (18.1)	
*No*	9,455 (83.5)	3,198 (85.1)	3,103 (83.6)	3,154 (81.9)	
LGA, n (%)					<0.001
*Yes*	1,522 (13.4)	391 (10.4)	484 (13.0)	647 (16.8)	
*No*	9,797 (86.6)	3,366 (89.6)	3,228 (87.0)	3,203 (83.2)	

GDM – gestational diabetes mellitus, LGA – large for gestational age, RC – remnant cholesterol

### Associations of RC levels and pre-pregnancy BMI with LGA risk

**Figure 1:**
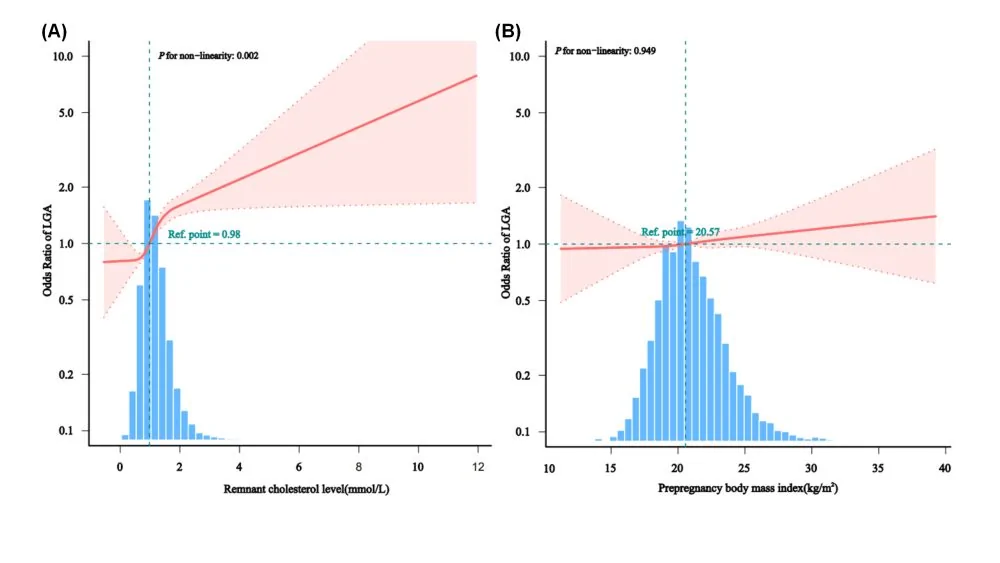
Dose-response relationship between remnant cholesterol (RC) level in first trimester, pre-pregnancy body mass index (BMI) and large for gestational age (LGA). Panel A. Remnant cholesterol. Panel B. Pre-pregnancy body mass index. The solid line and shadow part represents the unadjusted probability and adjusted 95% confidence intervals. Adjusted for maternal age, ethnicity, occupation, gravidity, parity, gestational age at delivery, infant sex, gestational weight gain, baseline, triglyceride, high-density lipoprotein cholesterol, and gestational diabetes mellitus. LGA – large for gestational age.

**Figure 2:**
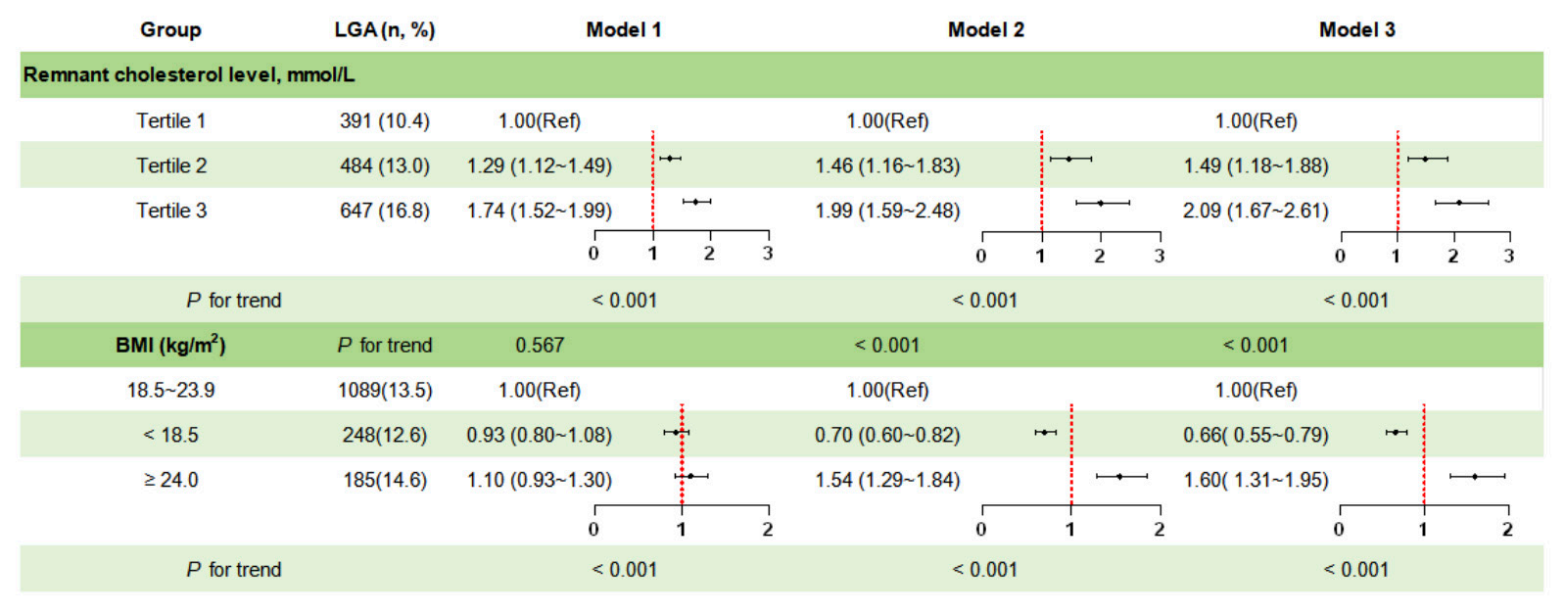
Associations between remnant cholesterol (RC) level and pre-pregnancy body mass index (BMI) with risk of large for gestational age (LGA). Model 1 was the crude model; Model 2 was adjusted for maternal age, ethnicity, occupation, gravidity, parity, gestational age at delivery, infant sex, and gestational weight gain; Model 3 further adjusted for baseline triglyceride, high-density lipoprotein cholesterol, and gestational diabetes mellitus. GDM – gestational diabetes mellitus, LGA – large for gestational age, RC – remnant cholesterol.

**Figure 3:**
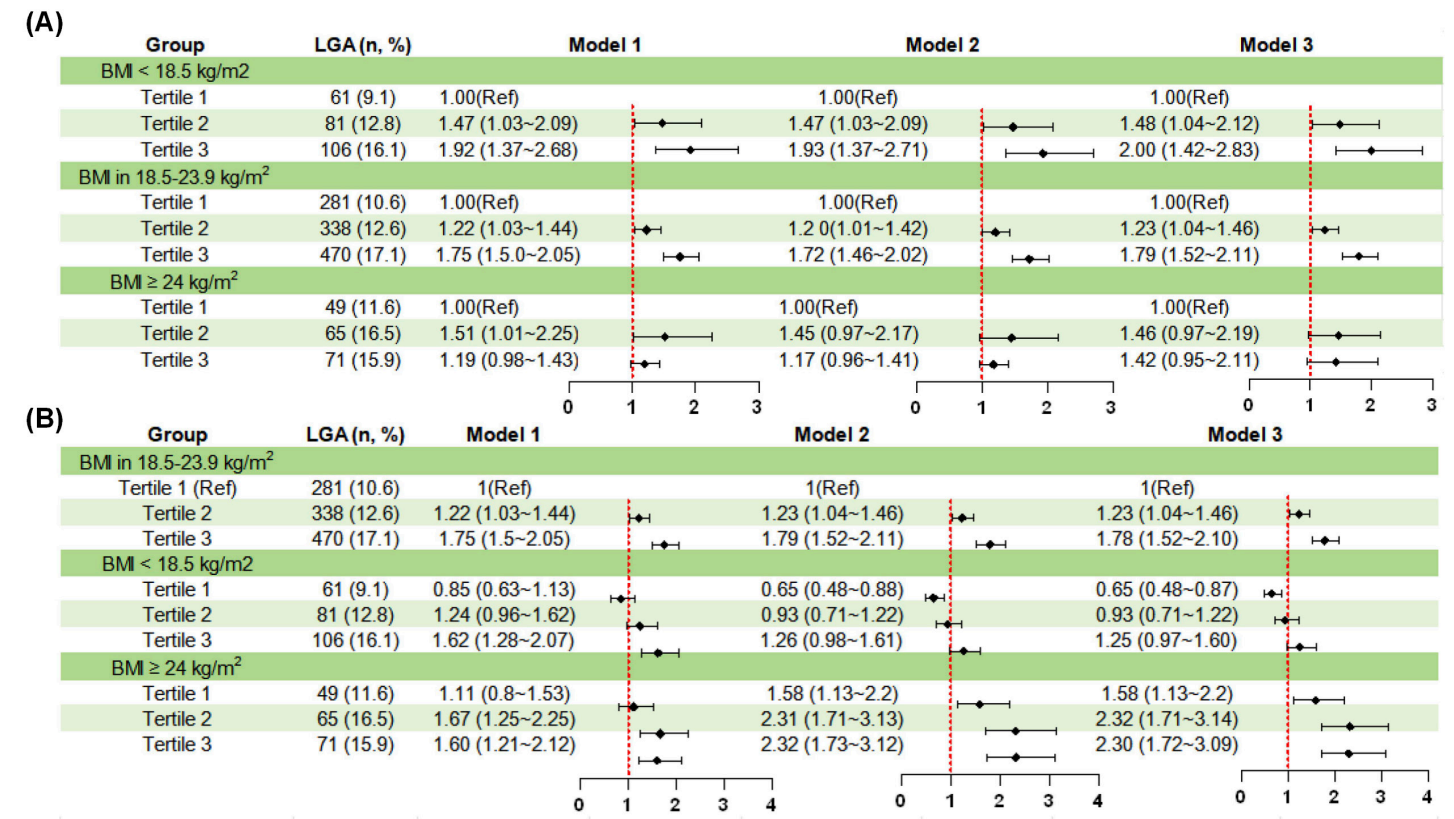
Interacting and joint effects of remnant cholesterol (RC) level and pre-pregnancy body mass index (BMI) on large for gestational age (LGA) risk. Panel A. Associations of RC level with risk of LGA by pre-pregnancy BMI. Panel B. Joint associations of RC level and pre-pregnancy BMI with risk of LGA. Model 1 was the crude model; Model 2 was adjusted for maternal age, ethnicity, occupation, gravidity, parity, gestational age at delivery, infant sex, and gestational weight gain; Model 3 further adjusted for baseline total cholesterol, triglyceride, high-density lipoprotein cholesterol, low-density lipoprotein cholesterol, and gestational diabetes mellitus. Wide confidence intervals in the underweight subgroup (BMI<18.5 kg/m^2^) reflect the smaller sample size; estimates should be interpreted with caution. BMI – body mass index, LGA – large for gestational age, RC – remnant cholesterol.

Restricted cubic spline regression revealed a nonlinear relationship between RC levels and LGA risk (*P*_for nonlinearity_ = 0.002), with an inflection point at 0.98 mmol/L. In contrast, a linear positive association was observed between pre-pregnancy BMI and LGA risk, with a cutoff value of 20.57 kg/m^2^ (**Figure 1**). The associations between RC levels, pre-pregnancy BMI, and the risk of LGA are presented in **Figure 2**. After full adjustment for potential confounders (Model 3), the adjusted OR for LGA in the highest RC tertile was 2.09 (95% CI = 1.67, 2.61) compared with the lowest tertile (*P*_for trend_<0.001). Similarly, compared with women of normal pre-pregnancy weight (BMI = 18.5–23.9 kg/m^2^), overweight/obese women (BMI≥24 kg/m^2^) had an adjusted OR of 1.60 (95% CI = 1.31, 1.95) for delivering an LGA infant (*P* for trend <0.001). Stratified analyses by pre-pregnancy BMI categories are presented in **Figure 3**, Panel A. Among women with normal pre-pregnancy weight (BMI = 18.5, 23.9 kg/m^2^), the adjusted ORs for LGA increased progressively with ascending RC tertiles. Compared with the lowest RC tertile, the ORs were 1.21 (95%CI = 1.04, 1.49) for tertile 2 and 1.80 (95% CI = 1.55, 2.20) for tertile 3. Although the underweight subgroup had a smaller sample size than the normal-weight group, the confidence intervals for these estimates remained informative, with the lower bound of the 95% CI for tertile 3 exceeding 1.0. These estimates should be interpreted with caution given the smaller sample size in the underweight subgroup. Among overweight/obese women, the association between RC tertiles and LGA risk did not reach statistical significance.

### Interaction and joint effects of pre-pregnancy BMI and RC levels

No significant multiplicative or additive interaction was observed between pre-pregnancy BMI and RC levels on LGA risk (**Table 2**). On the additive scale, the RERI was 0.120 (95% CI = −0.21, 0.49, *P* = 0.33), the AP was 0.05 (95% CI = −0.10, 0.20, *P* = 0.24), and the SI was 1.07 (95% CI = 0.77, 1.23, *P* = 0.55). On the multiplicative scale, the interaction term was not significant (RR = 0.90; 95% CI = 0.70, 1.04, *P* = 0.59). The joint association of pre-pregnancy BMI and RC levels with LGA is detailed in **Figure 3**, Panel B. Compared with women of normal weight (BMI = 18.5–23.9 kg/m^2^) and low RC levels (tertile 1), those who were overweight/obese (BMI≥24 kg/m^2^) and had high RC levels (tertile 3) had a 2.3-fold increased risk of LGA (adjusted OR = 2.30; 95% CI = 1.72, 3.09).

**Table 2:** Interactive effects of RC level and pre-pregnancy BMI on risk of LGA

Interactive items	Interactive effects					
	Model 1*		**Model 2†**		**Model 3‡**	
	**OR (95% CI)**	***P*-value**	**OR (95% CI)**	***P*-value**	**OR (95% CI)**	***P*-value**
Multiplicative effects	0.91 (0.73 ~ 1.14)	0.42	0.91 (0.73 ~ 1.14)	0.40	0.90 (0.70 ~ 1.04)	0.59
RERI	−0.09 (−0.38 ~ 0.2)	0.73	−0.09 (−0.38 ~ 0.2)	0.74	0.12 (−0.21 ~ 0.49)	0.33
AP	−0.06 (−0.24 ~ 0.13)	0.27	−0.06 (−0.25 ~ 0.13)	0.26	0.05 (−0.10 ~ 0.20)	0.24
SI	0.86 (0.56 ~ 1.33)	0.30	0.86 (0.55 ~ 1.34)	0.31	1.07 (0.77 ~ 1.23)	0.55

AP – proportion attributable to interaction, BMI – body mass index, CI – confidence interval, LGA – large for gestational age, OR – odd ratio, RC – remnant cholesterol, RERI – relative excess risk due to interaction, SI – synergy index

*Model 1 was the crude model.

†Model 2 was adjusted for maternal age, ethnicity, occupation, gravidity, parity, gestational age at delivery, infant sex, and gestational weight gain.

‡Model 3 further adjusted for baseline, triglyceride, high-density lipoprotein cholesterol, and gestational diabetes mellitus.

### Mediation analyses of BMI and RC level on the risk of LGA

Mediation analysis indicated that GDM significantly mediated the association between RC levels and LGA risk, accounting for 0.91% of the total effect. GDM also significantly mediated the relationship between pre-pregnancy BMI and LGA, with a mediation proportion of 1.15% (Table S1 in the **Online Supplementary Document**).

### Population attributable fraction

The population attributable fraction (PAF) was calculated to estimate the proportion of LGA cases attributable to high pre-pregnancy BMI (≥24 kg/m^2^) and elevated RC levels (≥0.98 mmol/L). High pre-pregnancy BMI accounted for 6.69% of LGA cases (n = 102), while high RC levels accounted for 19.68% of cases (n = 299) in this population (Table S2 in the **Online Supplementary Document**).

## DISCUSSION

This study aimed to investigate the independent and joint effects of first-trimester RC level and pre-pregnancy BMI on the risk of LGA infants, as well as to quantify their population-attributable fractions. Additionally, the mediating role of GDM in these associations was explored. Our findings provide a comprehensive understanding of how these metabolic factors intersect to influence foetal overgrowth, with important implications for clinical and public health practice.

Our results confirm the well-established association between elevated pre-pregnancy BMI and increased LGA risk [24–26]. We identified a pre-pregnancy BMI>20.57 kg/m^2^ as a potential risk threshold. However, this cutoff was derived from a data-driven approach (restricted cubic spline) using our single-centre cohort and falls within the normal BMI range according to Chinese classification standards. Therefore, this finding should be considered hypothesis-generating, and further external validation in independent cohorts is needed. This relationship is likely mediated by metabolic disturbances such as insulin resistance, which disrupts maternal glucose metabolism-a key driver of foetal overgrowth according to the Pedersen hypothesis [26,27]. While GDM was identified as a significant mediator in our study, its mediation proportion was modest (1.15%). This finding aligns with prior research suggesting GDM can mediate up to 16% of the effect of maternal obesity on LGA risk [28], and highlights that even with well-managed glucose levels, the underlying nutrient excess in overweight/obesity may promote foetal overgrowth [29,30]. These observations underscore the importance of preconception lifestyle interventions, including nutrition and physical activity, to mitigate both GDM risk and subsequent foetal overgrowth.

Furthermore, our study identifies elevated first-trimester RC levels as a significant and independent risk factor for LGA, with a nonlinear dose-response relationship (inflection point: 0.98 mmol/L). From a mechanistic perspective, RC may contribute to LGA risk through multiple interrelated pathways. It is important to distinguish RC from conventional lipid markers such as TG and non-high-density lipoprotein cholesterol (non-HDL-C). While TG mainly reflects the triglyceride load within lipoproteins, RC represents the cholesterol content carried specifically by remnant lipoproteins. Non-HDL-C is dominated by LDL-C and does not readily penetrate tissue barriers, while RC particles are smaller, more pro-inflammatory, and more capable of inducing endothelial dysfunction and placental inflammation. Several biological mechanisms may explain the observed association between RC and LGA. The particles of RC are small enough to infiltrate extravascular spaces, including pancreatic islets, where they can exert direct lipotoxic effects on β-cells. Accumulation of cholesterol in β-cells induces mitochondrial dysfunction, oxidative stress, and endoplasmic reticulum stress, ultimately leading to β-cell apoptosis and impaired insulin secretion [31,32]. Similarly, it has been established that RC activates pro-inflammatory pathways by stimulating nuclear factor-κB (NF-κB) signalling in macrophages and adipocytes, promoting the release of pro-inflammatory cytokines such as tumour necrosis factor-α (TNF-α) and interleukin-6 (IL-6). This low-grade chronic inflammation exacerbates systemic insulin resistance, a key metabolic disturbance in GDM pathogenesis [33–35]. Whether these mechanisms operate in pregnant women and directly contribute to LGA risk remains to be investigated. Third, RC may contribute to placental dysfunction by inducing endothelial inflammation and oxidative stress, although direct evidence in human pregnancy is lacking. This hypothesis is supported by studies of other lipoproteins, but future research is needed to determine whether RC specifically affects placental nutrient transport and foetal growth [36]. These distinct properties make RC a more sensitive marker for remnant particle-related metabolic risk than TG or non-HDL-C in the context of pregnancy and foetal overgrowth. Our mediation analysis showed that GDM mediated only 0.91% and 1.15% of the total effects of RC and pre-pregnancy BMI on LGA, respectively. Although these indirect effects reached statistical significance, likely due to the large sample size, the proportions are extremely small and of negligible practical importance. This finding indicates that GDM explains only a trivial fraction of the associations, and that the vast majority of the effects operate through other pathways. These may include direct effects of lipid metabolism on placental nutrient transport, chronic inflammation, insulin resistance independent of overt GDM, and other unmeasured mechanisms. It is also worth noting that dietary patterns, particularly the consumption of refined carbohydrates and animal fats, which are common in rapidly urbanising Southeast China, were not assessed in this study. Such dietary factors may influence both RC levels and LGA risk, and could potentially confound or moderate the observed association.

Although RC and TG are both markers of triglyceride-rich lipoprotein (TRL) metabolism, they reflect fundamentally different components of these particles. TG quantifies the glyceride fatty acid content of all TRLs, whereas RC specifically measures the cholesterol content within remnant particles (VLDL-C + IDL-C + chylomicron remnant-C).

Contrary to our initial hypothesis, we found no significant synergistic (multiplicative) or additive interaction between pre-pregnancy BMI and RC levels on LGA risk. While both factors independently elevate risk, their combined effect does not exceed the sum of their individual effects. This suggests that RC and BMI may operate through partially overlapping metabolic pathways, particularly insulin resistance and inflammation, rather than distinct synergistic mechanisms. Nonetheless, a joint analysis revealed that individuals with both high BMI (≥24 kg/m^2^) and high RC (highest tertile) faced the greatest risk. That is three times higher than those with normal BMI and low RC. This indicates that concurrent assessment of both markers is crucial for identifying women at highest risk, despite the absence of statistical interaction.

A key contribution of this study is the estimation of the PAF. We found that 6.69% and 19.68% of LGA cases in this cohort were attributable to high pre-pregnancy BMI (≥24 kg/m^2^) and elevated RC levels (≥0.98 mmol/L), respectively. These figures offer a direct public health metric of their impact. The PAF for high pre-pregnancy BMI in our cohort (6.69%) is lower than previously reported estimates from other populations, which typically range from 9.5 to 22.4% [30,37]. This discrepancy is likely attributable to differences in exposure prevalence and effect size across populations. In our cohort, the prevalence of high BMI was 11.2% with an adjusted OR of 1.64, whereas populations with higher PAFs have generally reported higher prevalences of overweight/obesity and/or larger effect sizes. To our knowledge, this is the first study to quantify the burden of LGA attributable to high RC levels, addressing a notable gap in the literature. Whether the RC threshold identified in this study (0.98 mmol/L) is generalisable to other ethnic groups remains unknown. This threshold was derived from a predominantly Han Chinese population in Southeast China. Lipid metabolism, genetic susceptibility, and dietary patterns differ substantially across populations. Therefore, this threshold should be considered population-specific, and external validation in multi-ethnic cohorts is warranted before clinical application beyond the study setting.

We recognise that the marked difference in PAFs between elevated RC and high pre-pregnancy BMI may appear to suggest a potentially greater population-level impact of lipid management. However, we would like to offer several considerations that, in our view, caution against directly comparing the magnitude of these two PAFs or concluding that lipid management should supersede weight management in LGA prevention. First, it is important to note that PAF is highly sensitive to the prevalence of the exposure in the study population. In our cohort, the prevalence of high pre-pregnancy BMI (≥24 kg/m^2^) was 11.2%, which is substantially lower than the prevalence of elevated RC (50.8%, defined by the inflection point of 0.98 mmol/L). Even if the true ORs for high BMI were comparable to or even greater than that for RC, a low exposure prevalence would inevitably result in a small PAF. Conversely, a moderately elevated RR for a relatively common exposure such as elevated RC can yield a large PAF. Therefore, we suggest that the observed discrepancy in PAFs primarily reflects differences in exposure prevalence rather than serving as a direct basis for comparing etiological strength. Second, the causal pathway linking pre-pregnancy BMI to LGA involves not only direct metabolic effects but also its well-recognised correlation with other lipid abnormalities, including elevated RC. Overweight and obese individuals frequently have higher RC levels; consequently, the independent PAF estimated for RC may partly capture effects that are biologically attributable to excess adiposity. We acknowledge that disentangling the independent *vs.* mediated contributions of BMI and RC remains methodologically challenging. Third, the clinical and public health implications of these two risk factors may differ. Weight management interventions, such as lifestyle modification before conception, are challenging to implement at the population level but offer broad health benefits beyond LGA prevention. Lipid management in the first trimester, while potentially more targeted, would require routine lipid screening, which is not yet standard in many antenatal care systems and safe pharmacological options during early pregnancy remain limited. We do not wish to overstate the immediate clinical applicability of lipid management based solely on the PAF estimates from a single cohort.

In light of these considerations, we do not interpret the PAF estimates as directly indicating that RC is ‘more important’ than BMI as a risk factor. Rather, we view them as complementary risk markers. Elevated RC may represent a more prevalent and potentially modifiable target in this specific population, but we believe that both factors should be considered in risk stratification. Future studies conducted in populations with higher BMI prevalence, and those incorporating direct validation of pre-pregnancy weight, would be valuable to determine whether the observed PAF difference persists or reflects underlying population characteristics.

Clinically, our findings suggest that risk assessment for foetal overgrowth should extend beyond traditional focus on overweight/obesity. Women with a pre-pregnancy BMI>20.57 kg/m^2^ showed increased risk in our cohort, challenging the conventional dichotomy of normal *vs.* overweight categories. Nevertheless, this data-driven threshold requires confirmation in independent populations before being considered for clinical use. Moreover, early-pregnancy lipid screening, particularly RC levels, should be considered in women with elevated BMI, as this may help identify those who would benefit from intensified monitoring or targeted lifestyle interventions (*e.g.* dietary modifications to reduce triglyceride-rich lipoprotein levels) to improve pregnancy outcomes.

The selection of the first trimester as the measurement window for RC was deliberate and justified on both physiological and clinical grounds. Physiologically, first-trimester lipid levels most closely approximate the pre-pregnancy metabolic baseline, minimising the confounding effects of pregnancy-induced physiological hyperlipidaemia and insulin resistance. By measuring RC in the first trimester, we capture the individual's underlying metabolic milieu that precedes and potentially sets the trajectory for pregnancy-associated lipid dysregulation, consistent with the developmental origins of health and disease framework. Clinically, early pregnancy offers a practical window for identifying high-risk women and implementing targeted interventions (*e.g.* dietary modification, physical activity) to mitigate LGA risk. Consistent with prior studies showing that first-trimester lipid profiles predict foetal growth independently of later metabolic changes, our data demonstrate a significant dose-response association between first-trimester RC levels and LGA risk.

Several limitations must be acknowledged. First, the observational design precludes causal inference; future randomised controlled trials or mechanistic studies are needed to establish causality. Second, although we adjusted for major confounders, unmeasured factors such as detailed dietary data or genetic predispositions may influence the results. In particular, dietary patterns especially the consumption of refined carbohydrates and animal fats common in rapidly urbanising Southeast China, were not assessed in this study. Such dietary factors are likely to influence both RC levels and LGA risk, and could potentially confound or moderate the RC-LGA association. Future studies should incorporate comprehensive dietary assessments to better understand these relationships. Third, RC was calculated indirectly rather than measured directly, which may introduce measurement error, particularly in pregnancy when lipoprotein metabolism is altered. Fourth, our single-centre cohort from a large tertiary hospital in Southeast China may introduce selection bias. Tertiary hospital populations typically have disproportionately higher rates of advanced maternal age, comorbidities (*e.g.* GDM, chronic hypertension), and greater socioeconomic heterogeneity compared with the general pregnant population. This selection is particularly relevant to our population attributable fraction (PAF) estimates, because PAF is directly influenced by the prevalence of exposure in the study sample. Specifically: for high pre-pregnancy BMI (≥24 kg/m^2^), the prevalence in our cohort was relatively low, which may lead to an underestimation of the true PAF at the general population level. For elevated RC (≥0.98 mmol/L), if our tertiary hospital sample over-enrols women with metabolic risk factors (*e.g.* older age, higher BMI, or abnormal lipid profiles), the observed RC prevalence may be higher than in the general obstetric population, potentially overestimating the PAF for RC. Therefore, the PAFs reported here (2.52% for high BMI and 19.68% for elevated RC) should be interpreted as estimates derived from a hospital-based high-risk cohort rather than as nationally representative figures. External validation in community-based or multi-centre population-based cohorts is needed to confirm the generalisability of these PAF estimates. Fifth, pre-pregnancy BMI was calculated using self-reported pre-pregnancy weight, which is subject to underreporting bias. It is well documented that women, particularly those with overweight or obesity, tend to underreport their weight, leading to systematic misclassification of some higher-BMI individuals into lower BMI categories [38,39]. This non-differential misclassification typically biases effect estimates toward the null, potentially attenuating the observed association between pre-pregnancy BMI and LGA risk. We did not have objectively measured pre-pregnancy weight for validation, which is a limitation of this large-scale cohort study. Consequently, the true association between pre-pregnancy BMI and LGA risk may be stronger than reported here. Future studies should incorporate objectively measured or medically recorded pre-pregnancy weight whenever possible to avoid this bias. Six, the generalisability of our findings is substantially limited by the single-centre design and the highly homogeneous study population. More than 98% of participants were of Han ethnicity, and all were recruited from one tertiary maternity hospital in Fujian Province, Southeast China. Although the sample size is large, women in our cohort may differ systematically from those receiving care in other regions, health systems, or with different ethnic and dietary backgrounds. Therefore, our findings, including the RC threshold of 0.98 mmol/L and the BMI threshold of 20.57 kg/m^2^, should not be directly generalised to other populations without external validation. Future large-scale, multi-ethnic prospective studies with direct RC measurement, comprehensive dietary assessment, and representation from diverse health systems are needed to determine the reproducibility and generalisability of our results.

## CONCLUSIONS

In summary, this study demonstrates that elevated pre-pregnancy BMI and high first-trimester RC levels are independent risk factors for LGA infants. Conversely, lower pre-pregnancy BMI and RC levels are associated with a decreased risk of LGA. Although GDM reached statistical significance as a mediator in these relationships, the proportions mediated were extremely small, indicating that GDM explains only a negligible fraction of the associations and that most of the effects operate through other pathways.

## Additional material


Online Supplementary Document

## Data Availability

**Data availability:** Data described in the manuscript will be made available upon request.
