# Supplementary material for: Association of pre-pregnancy body mass index and first-trimester remnant cholesterol level with risk of large for gestational age
Source: J Glob Health. 2026 Sep 4;16:04315. doi: 10.7189/jogh.16.04315 (PMC13543245; doi:10.7189/jogh.16.04315)

Supplement to: Lihua Lin, Sisi Zhang, Juan Lin, Xiaomei Wang. Association of pre-pregnancy body mass index and first-trimester remnant cholesterol level with risk of large for gestational age. J Glob Health. 2026;16:04315.

Supplementary Table S1 Mediating effects of GDM on the association between pre-pregnancy BMI and RC with LGA.

Model	Variable	Total effect(95%CI)	Natural direct effect(95%CI)	Natural indirect effect(95%CI)	Mediated proportion,%
**Model 1**	BMI	0.04533***	0.04585***	-0.00052*	1.15
**Model 2**	RC	0.05029***	0.04983***	0.000459***	0.91

****P*<0.001;**P*<0.05.

Model 1: Exposure = pre-pregnancy BMI , Mediator = GDM, Outcome = LGA.

Model 2: Exposure = RC, Mediator = GDM, Outcome = LGA.

Abbreviations: GDM, gestational diabetes mellitus; 95% CI, 95% confidence interval; RC:remnant cholesterol;LGA, large for gestational age; BMI, body mass index.

Supplementary Table S2. Estimated burden of rRC level and pre-pregnancy BMI attributable to fetal LGA.

	Number of total LGA	Attributed number of LGA	PAF(%)
BMI≥20.57	1522	771	2.52
RC≥0.98	1522	904	19.68

Abbreviations: PAF, population-attributable fraction;LGA, large for gestational age; BMI, body mass index;RC,remnant cholesterol.

Figure S1.

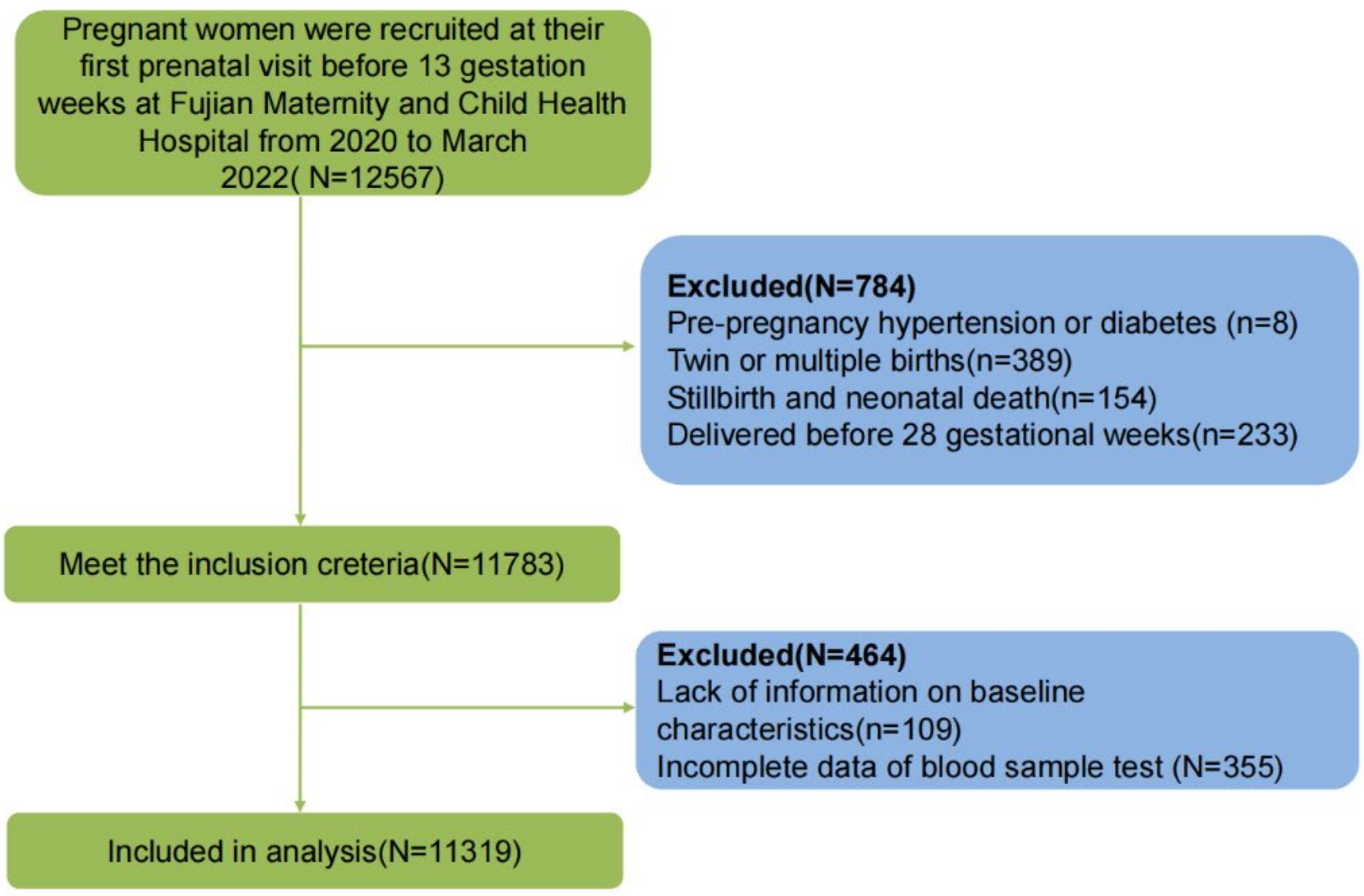

Figure S2.

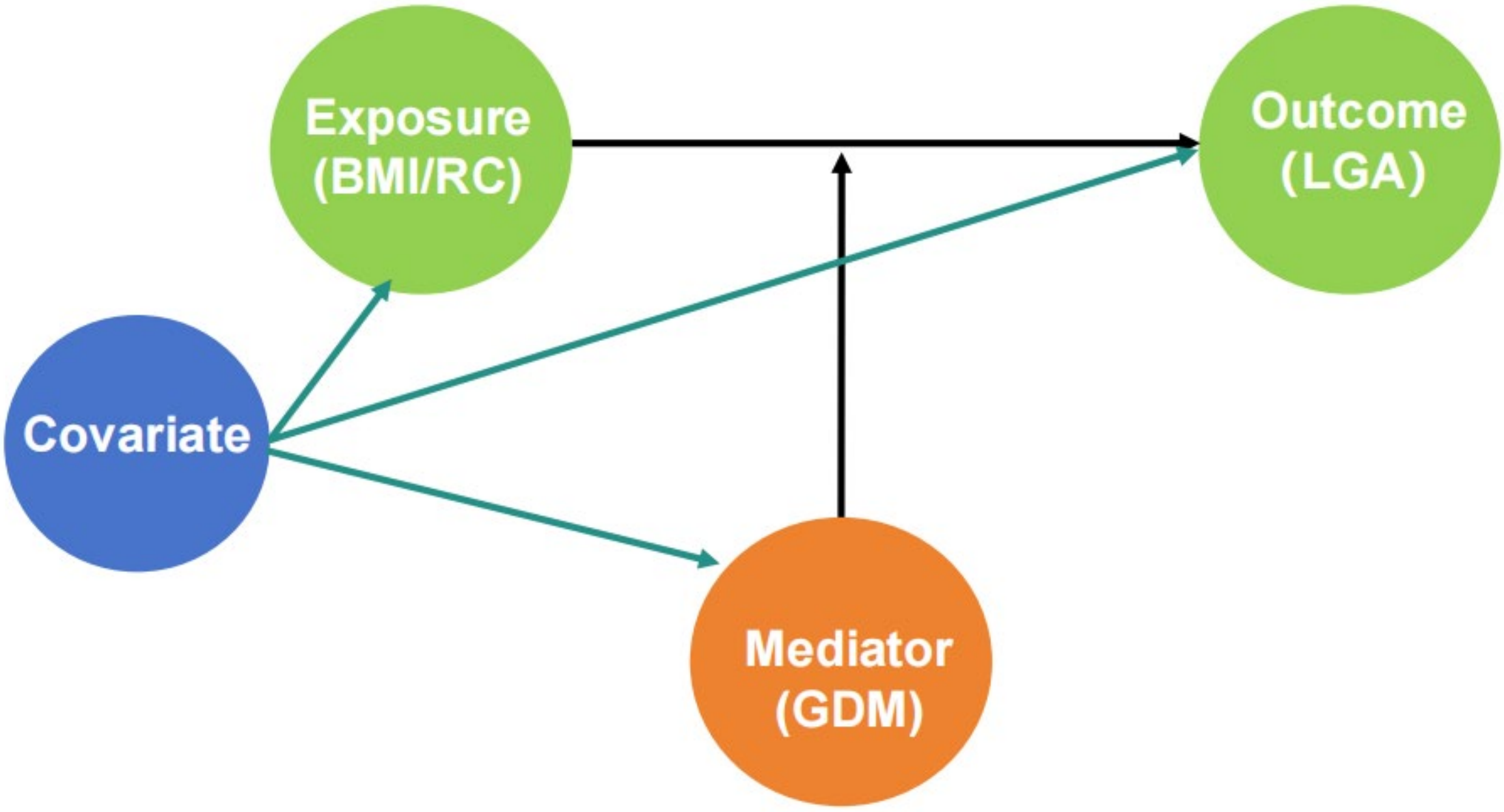

Supplement: Supplementary file 1 [file jogh-16-04315-s001.pdf]
